# A Lipopolysaccharide Synthesis Gene *rfaD* from *Mesorhizobium huakuii* Is Involved in Nodule Development and Symbiotic Nitrogen Fixation

**DOI:** 10.3390/microorganisms11010059

**Published:** 2022-12-25

**Authors:** Yuan Liu, Ye Lin, Ning Guan, Yuting Song, Youguo Li, Xianan Xie

**Affiliations:** 1 State Key Laboratory of Agricultural Microbiology, College of Life Science and Technology, Huazhong Agricultural University, Wuhan 430070, China; 2 State Key Laboratory of Conservation and Utilization of Subtropical Agro-Bioresources, Guangdong Laboratory for Lingnan Modern Agriculture, Guangdong Key Laboratory for Innovative Development and Utilization of Forest Plant Germplasm, College of Forestry and Landscape Architecture, South China Agricultural University, Guangzhou 510642, China

**Keywords:** symbiotic nitrogen fixation, gram-negative bacteria, *Mesorhizobium huakuii* 7653R, lipopolysaccharide, *rfaD*

## Abstract

Rhizobium lipopolysaccharide (LPS) is an important component of the cell wall of gram-negative bacteria and serves as a signal molecule on the surface of rhizobia, participating in the symbiosis during rhizobia–legume interaction. In this study, we constructed a deletion mutant of ADP-L-glycerol-D-mannoheptosyl-6-exoisomerase (*rfaD*) of *Mesorhizobium huakuii* 7653R and a functional complementary strain. The results showed that the deletion of *rfaD* did not affect the free-living growth rate of 7653R, but that it did affect the LPS synthesis and that it increased sensitivity to abiotic stresses. The *rfaD* promoter-GUS reporter assay showed that the gene was mainly expressed in the infection zone of the mature nodules. The root nodules formation of the *rfaD* mutant was delayed during symbiosis with the host plant of *Astragalus sinicus*. The symbiotic phenotype analyses showed that the nodules of *A. sinicus* lost symbiotic nitrogen fixation ability, when inoculated with the *rfaD* mutant strain. In conclusion, our results reveal that the 7653R *rfaD* gene plays a crucial role in the LPS synthesis involved in the symbiotic interaction between rhizobia and *A. sinicus*. This study also provides new insights into the molecular mechanisms by which the rhizobia regulate their own gene expression and cell wall components enabling nodulation in legumes.

## 1. Introduction

The successful establishment of the mutualistic endosymbiosis between rhizobia and legumes results from mutual recognition and exchange of signal molecules [1,2]. Flavonoids secreted by the roots of legumes can activate the nodulation genes of rhizobia to synthesize lipochitooligosaccharides (Nod factors; NFs) [3,4]. Subsequently, NFs are sensed by the cell surface LysM receptor heteromer of leguminous plants, resulting in the root hair curling, plasma membrane invagination, and cell division to form the infection threads and root nodules [5,6,7]. The rhizobia type Ⅲ secretion system (T3SS) enables rhizobia symbiosis through the NFs-independent signaling pathway [8]. The type Ⅲ effectors secreted by T3SS, also known as nodulation outer proteins (nops), play an important role in determining symbiosis specificity [9]. Meanwhile, the establishment of a rhizobia symbiosis requires an intimate connection between two symbionts, and cell surface structures will be involved in the early interaction between the rhizobia and legumes. Rhizobia secrete a variety of glycans on their cell surfaces, including lipopolysaccharides (LPS), exopolysaccharides (EPS), capsular polysaccharides (KPS), and cycloglucans (CG) [10,11]. These polysaccharides and glucans are necessary for the successful establishment of rhizobia symbiosis, especially at the early infection stages, and also determine the symbiotic specificity in the rhizobia–legumes interactions [11,12,13,14,15].

LPS forms the outer leaflet of the outer membrane of gram-negative bacteria, and maintains the cell membrane permeability of bacteria, enabling them to survive in harsh environments [16,17]. The LPS of rhizobia is essential for the formation of infection threads and symbionts, but inhibits the defense responses of legumes to accommodate the rhizobia within the host cells [18,19,20]. In *Rhizobium* sp. NGR234, the *lpsB* encodes a glycosyl transferase acting early in synthesis with the core part of the LPS; the LPS synthesis of *lpsB* mutant was affected, which seriously affected the establishment of its symbiotic relationship with *Vigna unguiculata*. [21]. Moreover, the *lpsB* and *lpsE* are two key genes in *Sinorhizobium fredii* HH103 involved in the LPS biosynthesis; however, the corresponding mutants cause the changes in the LPS structure and early nodule senescence, thereby impairing the symbiosis between *S. fredii* HH103 and soybean [22]. In addition, two genes, *lpxA* (encoding acyl-ACP-UDP-N-acetylglucosamine O-acyltransferase) and *lpxE* (encoding lipid A 1-phosphatase) in *Rhizobium* sp. PRF81, participate in the lipid A biosynthesis and LPS modification, and the expression of these two genes can be induced by the exudates released from common bean seeds [23]. Moreover, the purified LPS of *Sinorhizobium meliloti* inhibits the alkalization and reactive oxygen species burst in the alfalfa cells suspension, but not in non-host tobacco, which may suppress pathogenic responses in the host alfalfa to promote the rhizobia symbiosis [24]. The role of some genes regulating the LPS synthesis in rhizobia–legume symbiosis has been investigated on the bacterial side; however, the function of rhizobia LPS during symbiosis is not fully understood.

The LPS of rhizobia is composed of lipid A, core oligosaccharides, and O-antigen polysaccharide chains [16]. It has a general structure similar to that of animal gram-negative pathogens [25]. The core oligosaccharides of the LPS can be further divided into the inner and outer cores [26]. The part of the inner core is composed of 3-deoxy-D-manno-oct-2-ulosonic acid (Kdo) and ADP-heptose residues, while the outer core is composed of hexoses and 2-acetoamido-2-deoxy-hexoses and offers the attachment site for the O-polysaccharide chain. Furthermore, the ADP-heptose is an essential part of the inner core of the LPS, which links the exterior of the LPS to the Kdo between Kdo2-lipidA and O antigen. The ADP-heptose is also required for maintaining outer membrane integrity and limiting permeability [16,26,27]. The biosynthesis of the LPS has been extensively studied in *Escherichia coli* [28]. Studies have shown that the synthesis process includes five catalytic steps required to generate ADP-L-glycero-D-mannose-heptose, a molecule necessary for the first heptose that can be transferred to Kdo [27,28]. The chromosomal *rfa* in *E. coli* operably encodes the enzymes required for the stepwise assembly of major core oligosaccharides [16]. Among these genes, the *rfaD* gene encodes an ADP-L-*glycero*-D-manno-heptose-6-epimerase, which catalyzes the conversion of ADP-D-*glycero*-D-mannose-heptose to ADP-L-glycero-D-mannose-heptose, and this is the last step in the synthesis pathway of ADP-L-glycerol-D-mannose-heptose [27]. However, the function of *rfaD* genes in the symbiosis between rhizobia and legumes remains elusive.

Chinese milk vetch (*Astragalus sinicus*) is a traditional green manure legume widely grown in southern China. It can be used for improving the fertility of paddy fields, and can also be used as animal forage and nectar source plants [29]. *Mesorhizobium huakuii* 7653R is a Gram-negative bacterium which was isolated from the indeterminate root nodules of *A. sinicus* [30]. The symbiotic relationships of *A. sinicus* and *M. huakuii* 7653R have been extensively investigated [31,32,33]; moreover, the *M. huakuii* 7653R genome contains a single copy of the *rfaD* gene [34]. However, the function of *rfaD* in the symbiosis between *M. huakuii* 7653R and *A. sinicus* remains unknown.

In this study, we characterized the roles of the *rfaD* gene from *M. huakuii* 7653R. By constructing the *rfaD* deletion mutant, the phenotypes showed that the *rfaD* mutation affected the LPS synthesis and the host plants lost nitrogen fixation ability. The in situ gene expression analysis showed that the *rfaD* was mainly expressed in the infection zone of nodules of *A. sinicus*. Further observation of early infection events found that the nodule formation in the *rfaD* mutant was relatively delayed when compared with that in the wild type. Taken together, our findings revealed that the *rfaD* gene is required for rhizobia infection and nodulation in *A. sinicus*, and additionally provides new insights into the molecular mechanism by which the rhizobia orchestrate their own genes and cell wall components in the establishment of endosymbiosis in the roots of legumes.

## 2. Materials and Methods

### 2.1. Bacterial Strains and Plant Material and Growth Conditions

The bacterial strains used in this study are listed in Supplemental Table S1. Wild-type *M. huakuii* 7653R, and strains of 7653RΔ*rfaD*, 7653RΔ*rfaD*-C, 7653RΔ*rfaD*-GFP and 7653R pRG960-*rfaD* derived from 7653R were grown on tryptone yeast (TY) medium at 28 °C. *Escherichia coli* DH5a and S17-1 were cultured on Luria–Bertani (LB) medium at 37 °C. The following concentrations of antibiotics were used for the above strains: streptomycin, 100 μg mL^−1^; kanamycin, 50 μg mL^−1^; gentamycin, 25 μg mL^−1^; ampicillin, 100 μg mL^−1^; spectinomycin, 100 μg mL^−1^; tetracycline, 12.5 μg mL^−1^.

### 2.2. Symbiotic Phenotype Evaluation

*Astragalus sinicus* was used for symbiotic phenotype analysis. The seeds were surface-sterilized in 75% (*v*/*v*) ethanol for 10 min, washed six times with sterile water for 1 min each, and then treated with 5% sodium hypochlorite for 10 min, followed by 5–7 additional washes in sterile water [31,35]. To synchronize the germination, the sterilized seeds were immersed in distilled water for 18–24 h, and then placed on the sterile plates containing 0.5% sucrose and 0.55% agar. Seedlings were grown under a 16-h/8-h light/dark cycle at 25 °C/18 °C. After 5 days, the plantlets were transferred into sterile sand, and inoculated with 1 mL corresponding rhizobial culture at OD_600_ = 0.2 and watered with Fahraeus nitrogen-free plant nutrient solution [35]. At 28 days post-inoculation (dpi), the symbiotic plants were harvested, and the symbiotic phenotypes were examined.

### 2.3. Construction of the Deletion Mutants and Functional Complement Strains

**Figure 1:**
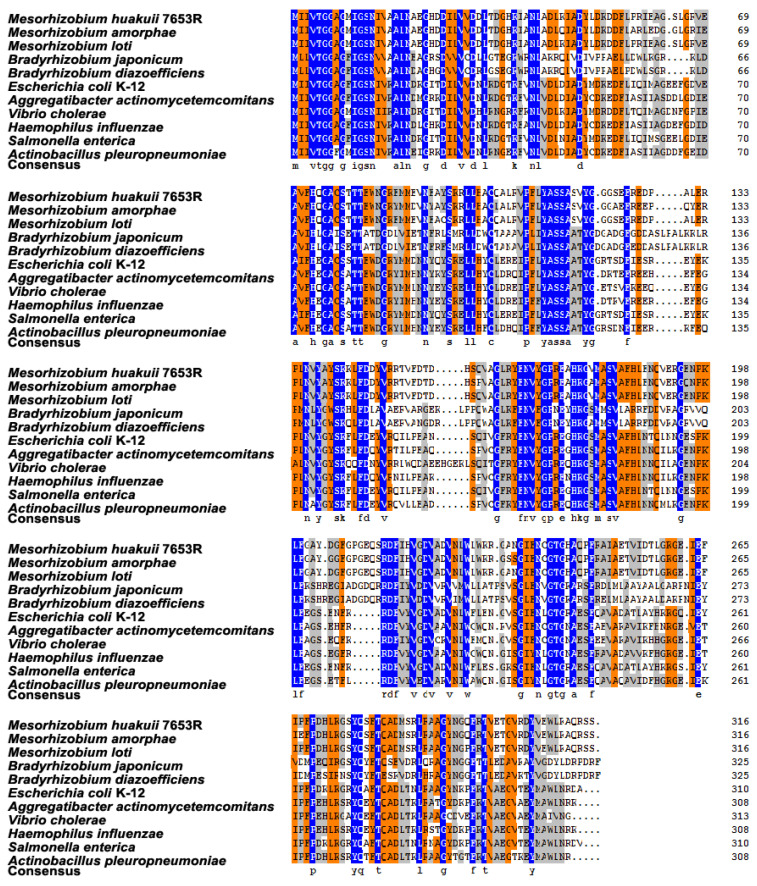
Multiple amino acids sequence alignments of the RfaD proteins from various bacteria species. Sequence alignments of the representatives of RfaD proteins from *Mesorhizobium*, *Bradyrhizobium* and some pathogens. Conserved residues are highlighted in blue.

In order to explore the biological function of *rfaD* in rhizobia symbiosis, the *cre-lox* system was used to construct the in-frame 7653R *rfaD* gene deletion mutant [36]. The fragments containing the flanking sequences of the 5′- and 3′-terminal coding regions of *rfaD* were amplified and cloned into the *Acc65*Ⅰ/*Nde*Ⅰand *Apa*Ⅰ/*Age*Ⅰmultiple cloning sites of pCM351. The recombinant plasmid was verified by PCR and Sanger sequencing, and transformed into *E. coli* S17-1, then conjugated into *M. huakuii* 7653R by bi-parental mating. To eliminate the gentamicin resistance gene flanked by the *loxP* site, the plasmid pCM157 carrying the *Cre* gene was introduced into the 7653RΔ*rfaD*::Gm by bi-parental conjugation. The transconjugants sensitive to gentamicin were screened, and the strains sensitive to tetracycline were then selected. The candidate strains were further confirmed by PCR and sequencing. To validate the correction of the obtained mutant strain, the genomic DNAs from wild-type and mutant strains were extracted, and amplified by PCR with the *rfaD*-MAP-F/R primers; the results showed that a large DNA fragment (3.1 Kb) was amplified using the genomic DNA from wild-type *M. huakuii* 7653R as a template, while a small DNA fragment (2.1 Kb) was specifically amplified using the *rfaD* mutant genomic DNA as a template (Figure 1). Meanwhile, these two genomic DNAs were also detected using the *rfaD*-ORF-F/R primers, and results showed that a 526 bp internal fragment of the *rfaD* ORF fragment was amplified using the genomic DNA from wild-type *M. huakuii* 7653R as a template, whereas no fragment was obtained using the *rfaD* mutant genomic DNA as a template (Figure S1). All the fragments obtained were subsequently sequenced, and the results showed the absence of the *rfaD* ORF in the 7653RΔ*rfaD* mutant strain. The strain with the *rfaD* deletion mutant was named as 7653RΔ*rfaD*.

To construct the recombinant plasmid carrying *rfaD*, the fragment containing the *rfaD* coding sequence and its promoter region (500 bp) was amplified and cloned into the *Sma*Ⅰ/*EcoR*Ⅰsites of the broad-host-range vector pBBR1MCS-5 [37]. The resulting construct pBBR1MCS-5-rfaD was transformed into the strain 7653RΔ*rfaD* by bi-parental mating. The strain obtained was named as 7653RΔ*rfaD*-C. The complementary strain was verified by PCR with the *rfaD*-MAP-F/R and *rfaD*-ORF-F/R primers (Figure S1); the DNA fragments represent the absence of the *rfaD* gene from the genome (2.1 kb) of *M. huakuii* 7653R and the presence of the *rfaD* gene in the complement plasmid pBBR1MCS-5-*rfaD* (526 bp), respectively. The gene-specific primers used for gene amplification in this study are listed in Supplemental Table S2.

### 2.4. LPS Extraction and Silver Staining

The LPS from the *M. huakuii* 7653R and 7653R*ΔrfaD* strains were extracted with an LPS extraction kit (iNtRON 17141) according to the manufactural procedures. The prepared LPS were added with 5 × SDS loading buffer and boiled, then separated by SDS-PAGE. The gel was fixed in 30% ethanol, 10% glacial acetic acid and 7 g/L periodate at 22 °C for 20 min, followed by being washed three times in sterile water. Subsequently, 1 g/L AgNO_3_ at 30 °C was added for 30 min for silver staining, with washing in sterile water for 10 s. Subsequently, 30 g/L Na_2_CO_3_ (4 °C), 0.02% formaldehyde color development for 20 min (fresh preparation before use) was added. Finally 10% glacial acetic acid was added to terminate the color reaction.

### 2.5. Plate Inhibition Test

The *M. huakuii* 7653R, 7653R*ΔrfaD*, and 7653R*ΔrfaD*-C strains were grown in liquid TY medium overnight to OD_600_ = 1.0. The cultures were mixed with the heated and melted TY solid medium sufficiently. The mixture was then poured into sterile plates. A sterile filter paper with a diameter of 5 mm was placed in the center of each plate. SDS (detergent), HCl (acid) and NaClO (oxidizing) were then dropped onto the filter paper. The plates were incubated at 28 °C for 3 days.

### 2.6. Construction of Promoter-GUS Reporter System and GUS Activity Assay

A 500-bp of upstream fragment of the *rfaD* was chosen as the putative promoter region, and then obtained from *M. huakuii* 7653R genomic DNA by PCR using the primers *rfaD*-*P-F* and *rfaD*-*P-R*. The amplicon was cloned into the *Sma*Ⅰ and *Pst*Ⅰ sites of pGR960 to create the pGR960-rfaD-GUS construct [38]. The recombinant plasmid was transformed into the wild-type *M. huakuii* 7653R. The recombinant strain was then inoculated to the roots of *A. sinicus*.

The roots and nodules of the *A. sinicus* plants were harvested and washed with distilled water to enable us to detect the *GUS* expression. The histochemical staining of GUS activity in the roots and nodules was performed according to the previous descriptions [39]. Roots and nodules were stained with the GUS buffer (0.1 M sodium phosphate buffer pH 7.0, 2 mM K_4_Fe (CN)_6_, 2 mM K_4_Fe (CN)_6_, 0.1% Triton X-100, 10 mM EDTA, 0.5 mg mL^−1^ X-Gluc dissolved in Dimethylformamide). Samples were incubated at 37 °C for 10 h in darkness and then washed with 70% (*v*/*v*) ethanol. The root and nodule sections were observed with a light microscope (BX51, Olympus, Japan) to visualize the spatial GUS activity.

### 2.7. Observation and Quantification of Early Infection Events

The wild-type 7653R and mutant 7653RΔ*rfaD* were labeled with a GFP reporter gene from vector pMP2463 [40]. The *A. sinicus* seedlings were inoculated with the strain 7653RΔ*rfaD*-GFP or 7653R-GFP. The early infection events, including root hair curling, infection threads, and nodular primordia, were observed and quantified at 3, 5, and 7days post-inoculation using a fluorescence microscope (BX51, Olympus, Tokyo, Japan).

### 2.8. Nitrogenase Activity Measurement

The nitrogenase activity of nodules was determined using the acetylene reduction activity method as described previously [33,41]. Briefly, the nodules of *A. sinicus* plants were harvested at 28 dpi for nitrogenase activity analysis. For each group, nine *A. sinicus* plants were randomly selected for the measurement. The root systems of each three plants, including roots and nodules, were incubated with 2 mL of acetylene for 2 h at 28 °C. The amount of ethylene was detected by the gas chromatograph (East & West Analytical Instrument GC 4000A, Dongxi, Beijing, China).

### 2.9. Analysis of Histological Nodule Cross-Sections

The microscopic analysis was performed according to methods previously described [31]. For light microscopic analysis, some nodules were fixed in formaldehyde-acetic acid buffer and dehydrated. The treated nodules were embedded in paraffin and cut longitudinally. The slides were stained with the Toluidine blue O and observed with a light microscope (BX51, Olympus, Tokyo, Japan). For electron microscopic analysis, the other nodules were first fixed in 2.5% glutaraldehyde for 4 h, then immersed in 1% osmium tetroxide for 3 h, dehydrated, and finally embedded in the London resin white [42]. The ultrathin sections were observed with an electron microscope (H-7650; Hitachi, Tokyo, Japan).

### 2.10. Phylogenetic Analysis and Conserved Sequence Alignment

The phylogenetic analysis of the rfaD proteins from bacteria was performed with MEGA11 software [43]. The amino acid sequences of rfaD were searched through the BLASTp program (https://blast.ncbi.nlm.nih.gov/Blast.cgi, accessed on 8 May 2022). The multiple protein sequences were aligned using the ClustalW, and the aligned sequences were loaded into the MEGA11 to generate an unrooted phylogenetic tree. The evolutionary history was inferred using the neighbor-joining method. The conserved sequence alignment was completed with DNAMAN software.

### 2.11. Accession Numbers

The sequence data of this article can be found in the GeneBank according to the following accession numbers: *Mesorhizobium huakuii* 7653R rfaD (WP_038648977.1). The rfaD homologous sequences for phylogenetic analysis were provided as below: *Bradyrhizobium japonicum* (WP_014493906.1), *Bradyrhizobium diazoefficiens* (WP_200519224.1), *Bradyrhizobium sp.* WSM1743 (WP_027576844.1), *Bradyrhizobium shewense* (WP_091959050.1), *Bradyrhizobium elkanii* (WP_028163631.1), *Bradyrhizobium lablabi* (WP_079543334.1), *Escherichia coli* K-12 (HBP1552200.1), *Mesorhizobium amorphae* (WP_192183840.1), *Mesorhizobium loti* (WP_019860550.1), *Mesorhizobium muleiense* (WP_091594992.1), *Mesorhizobium intechi* (WP_143977314.1), *Mesorhizobium comanense* (WP_137934890.1), *Mesorhizobium australicum* (WP_015316155.1), *Aggregatibacter actinomycetemcomitans* (WP_205772981.1), *Vibrio cholerae* (BCN19765.1), *Haemophilus influenzae* (WP_005657299.1), *Salmonella enterica* (WP_079802599.1), *Actinobacillus pleuropneumoniae* (WP_005605737.1).

### 2.12. Statistical Analysis

Statistical significance was determined by an independent-sample *t* test and single-factor analysis of the variance method with SPSS16 software (SPSS, Chicago, IL, USA). Three biological replicates were set up for each experiment. The error bars in the figures represent the standard deviation of three biological replicates. The asterisks indicate means that are statistically different at * *p* < 0.05, ** *p* < 0.01 and *** *p* < 0.001.

## 3. Results

### 3.1. Identification of rfaD in the Genome of Mesorhizobium huakuii 7653R

**Figure 2:**
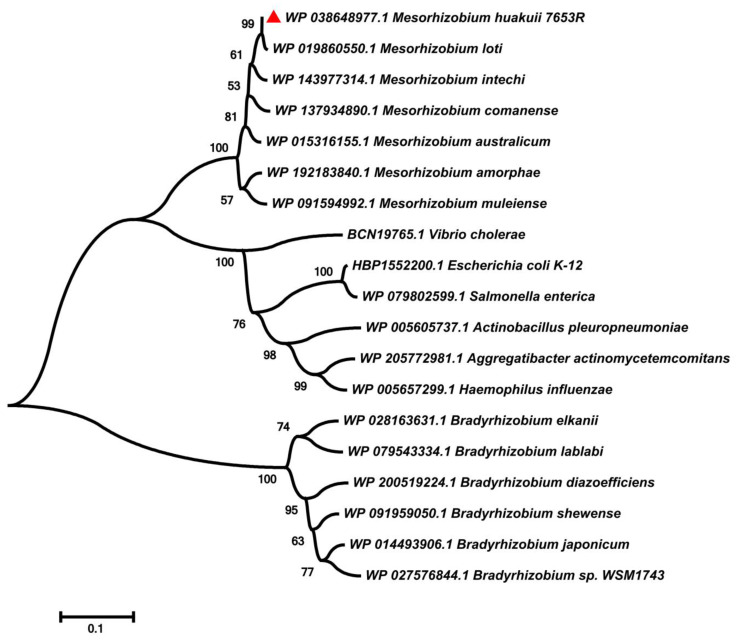
Phylogenetic analysis of RfaD homologues proteins among bacteria species. The phylogenetic relationship was inferred by MEGA11 software using the neighbor-joining method [43]. The evolutionary distance was computed using the *p*-distance method and presented in the number of amino acid differences per site. The branch of *M. huakuii* 7653R RfaD is marked with a red triangle. Tree scale, 0.1.

In the genome of *M. huakuii* 7653R, we found a single copy of the *rfaD* gene encoding an ADP-L-glycero-D-manno-heptose-6-epimerase [34]. Bioinformatics analysis showed that the *rfaD* gene had a 951 bp length of the open reading frame encoding 316 amino acids, with a predicted molecular weight of 35.01 kD and a theoretical pI of 5.69. Amino acid sequence alignment results showed that the rfaD proteins were relatively conserved across bacteria species, and the homologous proteins were conserved at the N-terminal regions (Figure 1). Phylogenetic analysis showed that the rfaD protein exists in a variety of Gram-negative bacteria, mainly in *Bradyrhizobium*, *Mesorhizobium* and pathogenic bacteria, but not in *Sinorhizobium* (Figure 2). This result indicated that the function of the *rfaD* in rhizobia-legume symbiosis may be species–specific. 

### 3.2. The 7653RΔrfaD Mutant Was Sensitive to Abiotic Stresses and Antibiotics

We initially examined the free-living growth of 7653R, 7653R*ΔrfaD*, and 7653R*ΔrfaD*-C strains. The result showed that the deletion of the *rfaD* gene had no significant effect on the growth of the 7653RΔ*rfaD* strain under free-living conditions (Figure S2). Subsequently, we examined the LPS synthesis in the wild-type 7653R and 7653RΔrfaD strains. The results showed that the LPS content of the *rfaD* mutant strain was clearly reduced when compared with that of the wild-type strain (Figure S2), indicating that the deletion of the *rfaD* affected the LPS synthesis of the 7653R strain. 

**Figure 3:**
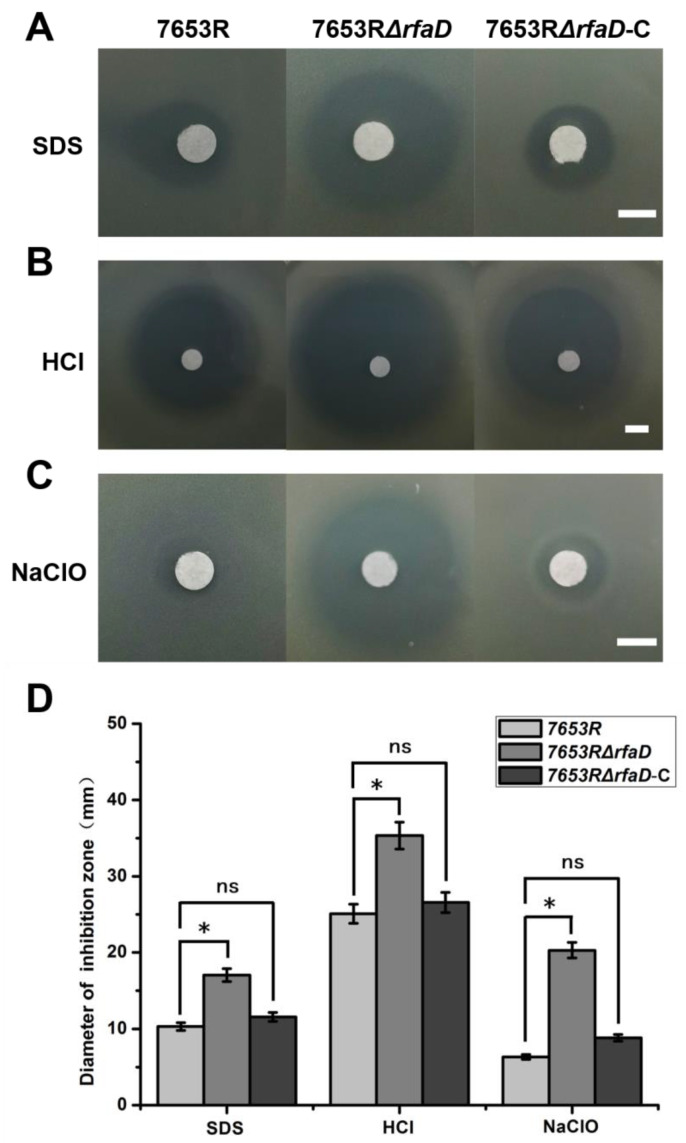
Inhibition zone determination of the wild-type 7653R, 7653RΔ*rfaD* and 7653RΔ*rfaD*-C strains. Two percent SDS (**A**), 5M HCl (**B**), NaClO (**C**) were used to detect the inhibition zone of wild-type 7653R, 7653RΔ*rfaD* and 7653RΔ*rfaD*-C. (**D**), the diameters of inhibition zones of the different indicated strains. Scale bars: 5 mm. Significance: * *p* < 0.05; ns, not significant; Student’s *t*-test. Data are shown as mean ± SD.

In order to further test whether the deletion of the *rfaD* would affect the stress resistance of the 7653R strain, we selected the reagents SDS (detergent), HCl (acidic) and NaClO (oxidant) to perform the plate inhibition zone experiments. The results showed that the mutation of the *rfaD* in 7653R led to a significant decrease in the stress resistance of rhizobia, whereas the functional complementary strain in response to abiotic stresses was comparable to that of the wild type (Figure 3). The previous report has shown that LPS is also resistant to polycationic antibiotics such as polymyxin B [44]. Therefore, we further detected the growth of *M. huakuii* 7653R, 7653RΔ*rfaD* and 7653RΔ*rfaD*-C strains on the TY plates in response to the polymyxin B addition. It was found that the 7653RΔ*rfaD* strain was more sensitive to the polymyxin B (50 μg/mL) than the wild-type strain (Figure S4). Collectively, these results suggest that the rhizobia LPS producing the gene *rfaD* plays a crucial role in the bacterial cell resistance to external stresses and antibiotics. 

### 3.3. The In-Situ Expression of the rfaD Gene in Nodules during Symbiosis

**Figure 4:**
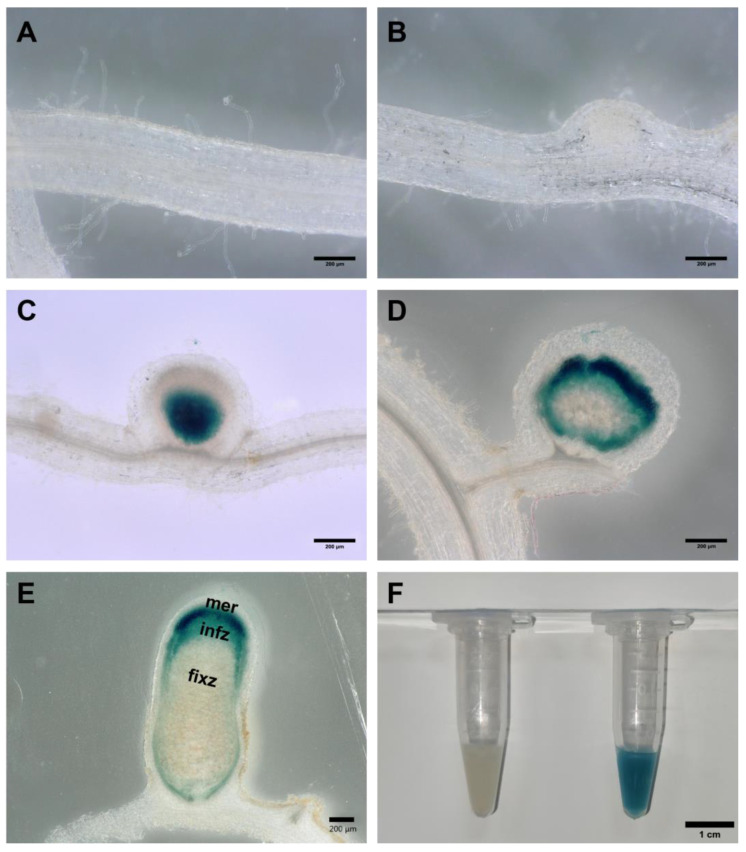
The in-situ expression pattern of the 7653R *rfaD* in *A. sinicus* roots during early infection and nodulation. (**A**–**E**), GUS staining of *A. sinicus* roots and nodules induced by *M. huakuii* 7653R carrying P_rfaD_-GUS. The *A. sinicus* roots were harvested at 3 dpi (**A**), 5 dpi (**B**), 9 dpi (**C**), 14 dpi (**D**) and 28 dpi (**E**). (**F**), GUS activity in the 7653R carrying empty plasmid pRG960 (left) and P_rfaD_-GUS fusions (right) in free-living conditions. Scale bars: 200 μm (**A**–**E**), 1 cm (**F**). mer, meristem zone; infz, infection zone; fixz, nitrogen fixation zone.

To investigate the in-situ spatial expression of the *rfaD* gene during nodulation in *A. sinicus*, we cloned the *rfaD* promoter, a 500 bp genomic DNA upstream fragment. This promoter fragment was fused with the GUS reporter in the pRG960 vector. The recombinant plasmid pRG960-rfaD was subsequently introduced into the wild-type *M. huakuii* 7653R, which was then inoculated into *A. sinicus* seedlings. The samples including roots and nodules were harvested separately at 3, 5, 9, 14 and 28 dpi for GUS staining analysis (Figure 4). At 3 dpi and 5 dpi, no GUS activity signals were detected at the curled root hairs and nodule primordia (Figure 4A,B). At 9 dpi, strong GUS activity was detected at the base of young nodules (Figure 4C). At 14 dpi and 28 dpi, GUS activity signals were found in the infection zone of mature nodules (Figure 4D,E). In addition, we also detected high GUS activity in the free-living bacterial cells of the 7653R strain carrying pRG960-rfaD; however, no GUS activity was detected in the *M. huakuii* 7653R strain with the empty vector pRG960 (Figure 4F). The spatiotemporal expression results showed that the *rfaD* gene was highly expressed in the infection zone of the mature nodules, suggesting that it may play a role in the infection process during symbiosis.

### 3.4. M. huakuii 7653R rfaD Gene Engages in the Early Infection Events during Rhizobia-A. sinicus Interaction

**Figure 5:**
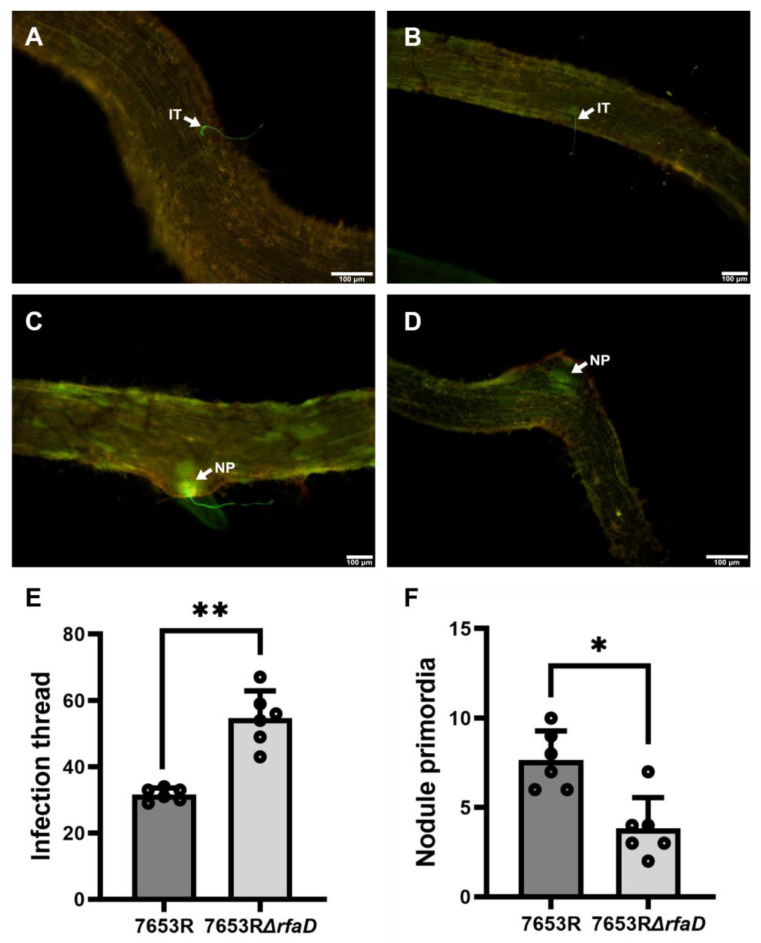
Observation and quantification of early infection events in *A. sinicus* roots inoculated with 7653R or 7653R*ΔrfaD*. (**A**–**D**), images of infection events of GFP-labeled 7653R (**A**,**C**) and 7653RΔ*rfaD* (**B**,**D**) at 7 dpi. A–B, infection thread; C–D, nodule primordium. E–F, Frequencies of infection threads (**E**) and nodule primordia (**F**) per root. The white arrows refer to infection thread and nodule primordium. IT, infection thread; NP, nodule primordium. Scale bars: 100 μm. Significance: * *p* < 0.05; ** *p* < 0.01; Student’s *t*-test. Data are shown as means ± SD.

To further explore the effect of the *rfaD* gene on the infection process during *M. huakuii* 7653R-*A. sinicus* interaction, we introduced the pMP2463-GFP plasmid into the *rfaD* mutant and 7653R strains. Therefore, the 7653RΔ*rfaD* mutant strain was marked by the GFP reporter gene, and could constitutively express the GFP protein. This transgenic strain was named 7653RΔ*rfaD*-GFP. Subsequently, we inoculated 7653RΔ*rfaD*-GFP into the roots of *A. sinicus* seedlings, while the wild-type 7653R-GFP was used as a control line. By visualizing the GPF signals during infection processes, we observed and quantified the rhizobia infection events during *M. huakuii* 7653R-*A. sinicus* interaction. In the *A. sinicus* roots with 7653R-GFP, the root hair curling was formed at the tips of root hairs, and infection threads were present in the curled root hairs, while the infection threads extended along the root hairs to the cortex, thereby forming a nodule primordium to develop the root nodules (Figure 5A,C). The early infection events in *A. sinicus* roots carrying the 7653RΔ*rfaD*-GFP were also observed and quantified at 7 dpi. The results showed that the number of curled root hairs and infection threads was significantly increased in the roots of *A. sinicus* inoculated with the 7653RΔ*rfaD*-GFP, while the number of nodule primordia was obviously decreased in this *rfaD* mutant when compared with the control strain 7653R-GFP (Figure 5B,D). Collectively, these findings suggest that the *M. huakuii* 7653R *rfaD* gene is involved in the early infection events during rhizobia-*A. sinicus* interaction and nodule formation.

### 3.5. M. huakuii 7653R rfaD Mutant Results in the Defective Nodules in A. sinicus

**Figure 6:**
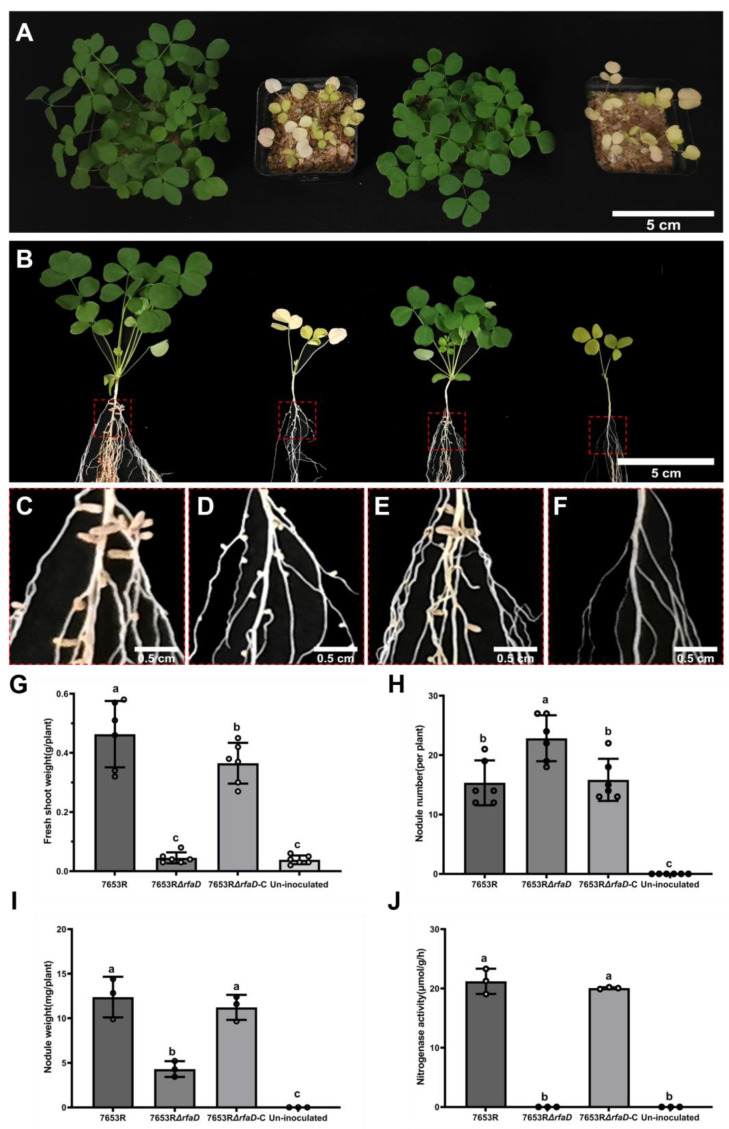
Symbiotic phenotypes of the *A. sinicus* plants inoculated with *M. huakuii* 7653R, 7653RΔ*rfaD*, or 7653RΔrfaD-C strains. *A. sinicus* plants were inoculated with the indicated strains and harvested at 28 dpi. A to F, the *A. sinicus* plants presented from the left to the right panels were inoculated with strain 7653R, 7653RΔ*rfaD*, 7653RΔ*rfaD*-C and without inoculation, respectively. (**A**), shoot growth performance; (**B**), whole-plant performance; (**C**–**F**), root nodule phenotypes, enlarged view of the red square; (**G**–**J**), fresh shoot weight, nodule numbers, nodule weights and the nitrogenase activity of plants inoculated with the strain 7653R, 7653RΔ*rfaD*, 7653RΔ*rfaD*-C, or not (un-inoculated), respectively. Scale bars = 5 cm (**A**,**B**), 2 mm (**C**,**E**) and 1 mm (**D**,**F**). Different letters are significantly different (*p* < 0.05) by one-way analysis of variance multiple comparisons.

To further investigate the role of the *rfaD* gene from *M. huakuii* 7653R in the processes of the rhizobia–legume symbiosis, we inoculated the strains of 7653R, 7653RΔ*rfaD*, or 7653RΔ*rfaD*-C into the roots of the host legume *A. sinicus*, while the plants without inoculation were used as the control lines. At 28 dpi, we harvested all the inoculated and non-inoculated plants. The *A. sinicus* plants inoculated with 7653RΔ*rfaD* exhibited a defective symbiotic phenotype: the biomass of the shoots of the plants decreased, the leaves turned yellow, and the morphology and color of the formed nodules were abnormal, being small white round nodules. However, the *A. sinicus* plants inoculated with the functional complementary strain exhibited normal growth and were almost similar to the 7653R wild-type plants; the shoot biomass was larger, the leaves were green, and the morphology and color of root nodules were normal with a pink color (Figure 6A–F). 

The quantitative analysis showed that, compared with the 7653R wild-type, the symbiotic phenotype of the *M. huakuii* 7653RΔ*rfaD* strain, the fresh weight of the shoot biomass and nodules were significantly reduced, whereas the number of root small nodules was significantly increased, and the nitrogenase activity of root nodules after being inoculated with 7653RΔrfaD was not detectable (Figure 6G–J). Additionally, combined with the results of early infection events, it is suggested that the lack of *rfaD* results there is a delay in the plant–*rfaD* mutant symbiosis, and that they finally form ineffective root nodules. On the other hand, there was no significant difference in the shoot biomass, root nodules, nodules number, or the nitrogenase activity of nodules between the 7653RΔ*rfaD*-C and the wild-type 7653R strains. These results indicated that the 7653RΔ*rfaD*-C strain restored the defective nitrogen fixation ability of the 7653RΔ*rfaD* mutant.

**Figure 7:**
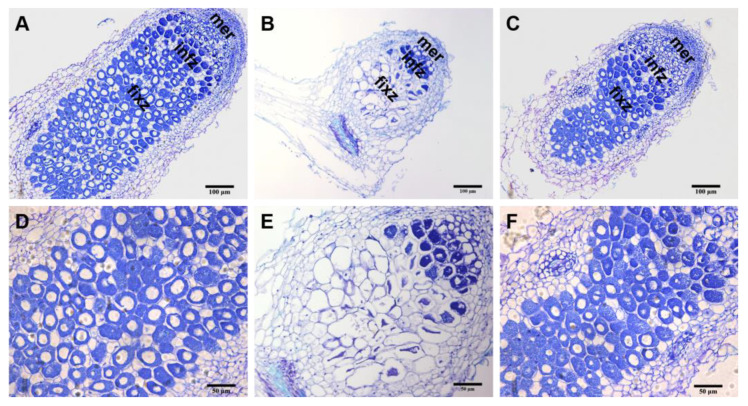
Observation of the nodule structures from *A. sinicus* roots inoculated with *M. huakuii* 7653R, 7653RΔ*rfaD*, or 7653RΔrfaD-C strains. (**A**–**F**), the paraffin sections of nodules from *A. sinicus* roots after staining with Toluidine blue O. A and D, inoculation with wild-type 7653R; (**B**,**E**), inoculation with 7653R*ΔrfaD* mutant; (**C**,**F**), inoculation with 7653R*ΔrfaD*-C. mer, meristem zone; infz, infection zone; fixz, nitrogen fixation zone. Scale bars: 100 μm (**A**–**C**) and 50 μm (**D**–**F**).

**Figure 8:**
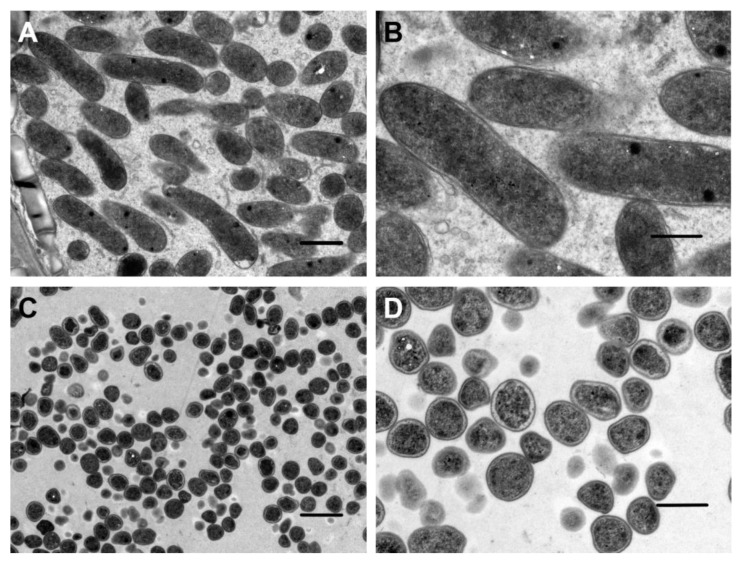
Observation of the ultrastructures of *A. sinicus* nodules generated by the *M. huakuii* 7653R and 7653R*ΔrfaD*. Transmission electron microscope images of the nodules from *A. sinicus* roots at 28 dpi. (**A**,**B**), inoculation with wild-type 7653R; (**C**,**D**), inoculation with 7653R*ΔrfaD*. Scale bars: 2 μm (**A**,**C**) and 1 μm (**B**,**D**).

To next dissect the structures of root nodules of the *A. sinicus*, we performed a histological analysis of the nodules from the 7653R, 7653R*ΔrfaD*, and 7653R*ΔrfaD*-C strains. The results showed that the nodules from the 7653RΔ*rfaD* mutant contained a considerable number of uninfected root cells, while only a small number of rhizobia cells were found in the infective zone of the nodules. In contrast, the nodules of plants inoculated with the 7653R wild-type or complement strain 7653R*ΔrfaD*-C contained large numbers of symbiotic cells with bacteroid (Figure 7). In order to further examine the effect of the *rfaD* deletion on the development of bacteroid, we observed the ultrastructure of wild-type and Δ*rfaD* mutant nodules by transmission electron microscopy. The results showed that the rhizobia in the wild-type nodules differentiated into normal elongated bacteroid surrounded by symbiosome membranes (Figure 8A,B). On the contrary, in the Δ*rfaD* mutant nodules, the differentiation in the bacteroid was abnormal; they were small spheres (Figure 8C,D).

Taken together, these findings reveal that the *rfaD* deletion impairs the normal differentiation in the bacteroid, resulting in the formation of defective nodules.

## 4. Discussion

The symbiotic nitrogen fixation between rhizobia and legumes is a delicate and complex process, which is involved in a large number of genes and chemical components from both symbionts [2,45]. Among them, the lipopolysaccharide (LPS) is an important component of the cell wall of gram-negative bacteria and plays an important role in the symbiosis between rhizobia and leguminous plants [10,18]. The LPS in *E. coli* was extensively studied [27,46,47,48], and some rhizobia also have the gene information of the LPS synthetic pathway, but the precise functions of LPS synthesis-related genes in the process of symbiotic nitrogen fixation remain elusive. It has been reported that in some Gram-negative bacteria, the deletion of the *rfaD* gene (encoding an ADP-L-glycero-D-manno-heptose-6-epimerase) will affect the biosynthesis of the inner core of the LPS [16,27]. Here, we focused on the *rfaD* gene in *M. huakuii* 7653R, by investigating its roles in LPS synthesis and stress resistance, its expression patterns during symbiosis and its roles in the early infection and nodulation.

In this study, we constructed a *rfaD* gene deletion mutant using the *cre-loxp* system and explored its functions in free-living growth and during symbiosis. Quantitative detection of the LPS extracted from 7653R*ΔrfaD* and wild-type 7653R strains showed that the deletion of the *rfaD* gene did directly lead to a lower level of LPS synthesis. Furthermore, the ability of the 7653R*ΔrfaD* strain to resist external stresses decreased significantly under free-living conditions. It is reasonably proposed that this strain sensitivity to stresses may have resulted from the lower LPS production of this *ΔrfaD* mutant. Because the LPS is an important component of the cell wall of Gram-negative bacteria, the very low amounts of LPS may change the permeability of the cell wall, thereby affecting the ability of bacteria to resist external stress. These results further indicate that the epimerase encoded by the *rfaD* converts the ADP-D-glycerol-β-D-mannoheptose into the ADP-L-glycerol-β-D-mannoheptose; this reaction is necessary for LPS synthesis [27]. Therefore, the ADP-L-glycerol-β-D-mannoheptose can participate in the synthesis of the inner core of the LPS. The core polysaccharide is connected to the other two important components of the LPS: lipid A and O antigen; thus, the defective synthesis of the core polysaccharide has an impact on the structure of the LPS.

LPS is also considered as one of the essential components in the symbiosis between rhizobia and legumes [19,49]. The mutant of the *rfaD* gene of *Azorhizobium caulinodans* ORS571, which forms nodules on the stems and roots of *Sesbania rostrata*, was more advanced than the wild-type strain, but the mutant nodules were ineffective [50]. In this work, the inoculated 7653R*ΔrfaD* into the roots of *A. sinicus*, the symbiotic phenotype analysis showed that the shoots of the symbiotic plants grew poorly, and the leaves were yellow in color (an indicator of lacking N). Furthermore, the development of root nodules was abnormal and defective: in the *ΔrfaD* mutant roots, nodules were small and white, and the nitrogenase activity of nodules was not detectable. This led us to think about and analyze the possible causes of these symbiotic phenotypes. Because of the deletion mutation of the *rfaD*, the structure or synthetic quantity of the LPS changed in the mutant strain, which further interfered with the balance of symbiosis and immunity during the interactions between the rhizobia and the host, thus affecting the normal release and survival of the rhizobia from the infection threads (ITs), and significantly reducing the number of infected plant cells in the root nodules; the mutant forms were therefore ineffective and white nodules. Furthermore, the enhanced sensitivity of the outer membrane of the rfaD mutant may affect the normal differentiation of the bacteroid, which leads to a loss of nitrogen fixation ability of the mutant nodules. The results indicated that the *rfaD* is an important gene in the process of symbiotic nitrogen fixation, suggestive of the activation of symbiotic nitrogen fixation by regulating the LPS synthesis. The previous studies reported that the LPS could regulate symbiotic nitrogen fixation at the multiple infection stages, such as the inhibition of plant defense responses during infection, and participation in the formation of infection threads [18,19,20]. Core polysaccharide and O-antigen of the LPS may change during development and symbiotic interaction [20]. The rhizobia LPS would become more hydrophobic during bacterial development, and would also change due to plant exudate [51,52,53]. Therefore, the LPS synthesis-related genes may play different roles in the autogenesis growth and symbiotic interaction. The results from the in-situ expression experiment showed that the *rfaD* gene was highly expressed in the infection zone of root nodules, but not in the early infection process. This result may be due to the low expression of the gene in the early stage of infection. Alternatively, it is predicted that the *rfaD* gene also functions in the infected zone at the late stage of nodulation, such as protecting bacterial cells in the nitrogen fixation zone as a barrier, or participating in the molecular information exchange at the symbiotic interface. The researches on *rfaD* in some pathogenic bacteria reported that the deletion of this gene would lead to a short-of-LPS-sugar chain, thereby reducing the adhesion of bacteria to the host cells and reducing the pathogenicity of bacteria [26,27]. Moreover, the observation on the 7653R*ΔrfaD* inoculated into *A. sinicus* roots found that the number of infection threads increased at the early stage of infection; this result may be caused by the decrease in the adhesion ability of the rhizobia 7653R*ΔrfaD* (due to the lower LPS synthesis) during the infection processes, resulting in a defective nodule development. In the process of symbiosis with the rhizobia, plants increase their nitrogen fixation ability by regulating the number of nodules [54,55]. However, the low number of nodule primordia in the 7653R*ΔrfaD* strain may be due to the lagging process of nodule formation. The above findings indicate that the *rfaD* gene plays a role in the infected zone after nodule formation, regulating nodule development and nitrogen fixation, although it is not highly expressed at the early stage of nodule formation.

In conclusion, the present study showed an ADP-L-glycerol-D-mannoheptosyl-6-exoisomerase coding gene, *rfaD* from *M. huakuii* 7653R. The expression, localization and function experiments revealed the *rfaD* engaged in the LPS synthesis required for nodulation during the *M. huakuii* 7653R-*A. sinicus* interaction. These new findings advance our understanding of the function of LPS synthesis-related genes from rhizobia in the establishment of endosymbiosis in legumes.

## Data Availability

National Center for Biotechnology Information (NCBI) at https://www.ncbi.nlm.nih.gov/.

## Supplementary Materials

The following supporting information can be downloaded at: https://www.mdpi.com/article/10.3390/microorganisms11010059/s1, Figure S1: Verification of the constructed M. huakuii 7653R *rfaD* deletion mutant (7653R*ΔrfaD*) [56]; Figure S2: Effects of 7653R *rfaD* on the rhizobia growth under free-living conditions; Figure S3: The LPS produced by wild-type 7653R and 7653R*ΔrfaD* mutant; Figure S4: Sensitivity of the 7653R, 7653R*ΔrfaD* and 7653RΔrfaD-C strains to the polymyxin B. Table S1: The strains and plasmids used in this study; Table S2: Primers used in this study.

## References

[1] Oldroyd G.E., Murray J.D., Poole P.S., Downie J.A. (2011). The rules of engagement in the legume-rhizobial symbiosis. Annu. Rev. Genet..

[2] Via V.D., Zanetti M.E., Blanco F. (2016). How legumes recognize rhizobia. Plant Signal. Behav..

[3] Hassan S., Mathesius U. (2012). The role of flavonoids in root-rhizosphere signalling: Opportunities and challenges for improving plant-microbe interactions. J. Exp. Bot..

[4] Wang D., Yang S., Tang F., Zhu H. (2012). Symbiosis specificity in the legume: Rhizobial mutualism. Cell. Microbiol..

[5] Madsen E.B., Madsen L.H., Radutoiu S., Olbryt M., Rakwalska M., Szczyglowski K., Sato S., Kaneko T., Tabata S., Sandal N. (2003). A receptor kinase gene of the LysM type is involved in legume perception of rhizobial signals. Nature.

[6] Madsen L.H., Tirichine L., Jurkiewicz A., Sullivan J.T., Heckmann A.B., Bek A.S., Ronson C.W., James E.K., Stougaard J. (2010). The molecular network governing nodule organogenesis and infection in the model legume *Lotus japonicus*. Nat. Commun..

[7] Oldroyd G.E. (2013). Speak, friend, and enter: Signalling systems that promote beneficial symbiotic associations in plants. Nat. Rev. Microbiol..

[8] Okazaki S., Kaneko T., Sato S., Saeki K. (2013). Hijacking of leguminous nodulation signaling by the rhizobial type III secretion system. Proc. Natl. Acad. Sci. USA.

[9] Sugawara M., Takahashi S., Umehara Y., Iwano H., Tsurumaru H., Odake H., Suzuki Y., Kondo H., Konno Y., Yamakawa T. (2018). Variation in bradyrhizobial NopP effector determines symbiotic incompatibility with Rj2-soybeans via effector-triggered immunity. Nat. Commun..

[10] Fraysse N., Couderc F., Poinsot V. (2003). Surface polysaccharide involvement in establishing the rhizobium-legume symbiosis. Eur. J. Biochem..

[11] Kawaharada Y., Kelly S., Nielsen M.W., Hjuler C.T., Gysel K., Muszyński A., Carlson R.W., Thygesen M.B., Sandal N., Asmussen M.H. (2015). Receptor-mediated exopolysaccharide perception controls bacterial infection. Nature.

[12] Becker A., Fraysse N., Sharypova L. (2005). Recent advances in studies on structure and symbiosis-related function of rhizobial K-antigens and lipopolysaccharides. Mol. Plant-Microbe Interact..

[13] Jones K.M., Kobayashi H., Davies B.W., Taga M.E., Walker G.C. (2007). How rhizobial symbionts invade plants: The Sinorhizobium-Medicago model. Nat. Rev. Microbiol..

[14] Janczarek M., Rachwał K., Marzec A., Grządziel J., Palusińska-Szysz M. (2015). Signal molecules and cell-surface components involved in early stages of the legume–rhizobium interactions. Appl. Soil Ecol..

[15] Chu H., Mazmanian S.K. (2013). Innate immune recognition of the microbiota promotes host-microbial symbiosis. Nat. Immunol..

[16] Raetz C., Whitfield C. (2002). Lipopolysaccharide endotoxins. Annu. Rev. Biochem..

[17] Maldonado R.F., Sá-Correia I., Valvano M.A. (2016). Lipopolysaccharide modification in Gram-negative bacteria during chronic infection. FEMS Microbiol. Rev..

[18] Lerouge I., Vanderleyden J. (2002). O-antigen structural variation: Mechanisms and possible roles in animal/plant-microbe interactions. FEMS Microbiol. Rev..

[19] Scheidle H., Gross A., Niehaus K. (2005). The Lipid A substructure of the Sinorhizobium meliloti lipopolysaccharides is sufficient to suppress the oxidative burst in host plants. New Phytol..

[20] Xiang Q., Wang J., Qin P., Adil B., Xu K., Gu Y., Yu X., Zhao K., Zhang X., Ma M. (2020). Effect of common bean seed exudates on growth, lipopolysaccharide production, and lipopolysaccharide transport gene expression of Rhizobium anhuiense. Can. J. Microbiol..

[21] Ardissone S., Noel K.D., Klement M., Broughton W.J., Deakin W.J. (2011). Synthesis of the flavonoid-induced lipopolysaccharide of Rhizobium Sp. *strain NGR*234 requires rhamnosyl transferases encoded by genes rgpF and wbgA. Mol. Plant-Microbe Interact..

[22] Margaret I., Lucas M.M., Acosta-Jurado S., Buendía-Clavería A.M., Fedorova E., Hidalgo Á., Rodríguez-Carvajal M.A., Rodriguez-Navarro D.N., Ruiz-Sainz J.E., Vinardell J.M. (2013). The Sinorhizobium fredii HH103 lipopolysaccharide is not only relevant at early soybean nodulation stages but also for symbiosome stability in mature nodules. PloS ONE.

[23] Oliveira L.R., Rodrigues E.P., Marcelino-Guimarães F.C., Oliveira A.L., Hungria M. (2013). Fast induction of biosynthetic polysaccharide genes lpxA, lpxE, and rkpI of Rhizobium sp. strain PRF 81 by common bean seed exudates is indicative of a key role in symbiosis. Funct. Integr. Genom..

[24] Albus U., Baier R., Holst O., Pühler A., Niehaus K. (2001). Suppression of an elicitor-induced oxidative burst reaction in Medicago sativa cell cultures by *Sinorhizobium meliloti* lipopolysaccharides. New Phytol..

[25] Carlson R.W., Forsberg L.S., Kannenberg E.L. (2010). Lipopolysaccharides in Rhizobium-legume symbioses. Sub-Cell. Biochem..

[26] Chang P.C., Wang C.J., You C.K., Kao M.C. (2011). Effects of a HP0859 (rfaD) knockout mutation on lipopolysaccharide structure of Helicobacter pylori 26695 and the bacterial adhesion on AGS cells. Biochem. Biophys. Res. Commun..

[27] Kuo C.J., Chen J.W., Chiu H.C., Teng C.H., Hsu T.I., Lu P.J., Syu W.J., Wang S.T., Chou T.C., Chen C.S. (2016). Mutation of the enterohemorrhagic *Escherichia coli* core LPS biosynthesis enzyme RfaD confers hypersusceptibility to host intestinal innate immunity in vivo. Front. Cell. Infect. Microbiol..

[28] Valvano M.A., Messner P., Kosma P. (2002). Novel pathways for biosynthesis of nucleotide-activated glycero-manno-heptose precursors of bacterial glycoproteins and cell surface polysaccharides. Microbiology.

[29] Cho H.J., Yong X., Murooka Y. (1995). Formation of adventitious shoots and plant regeneration by culture of cotyledon segment in Astragalus sinicus (Chinese Milk Vetch). Plant Tissue Cult. Lett..

[30] Chen H.K., Shu M.K. (1944). Note on the root-nodule bacteria of Astragalus sinicus L.. Soil Sci..

[31] Lei L., Chen L., Shi X., Li Y., Wang J., Chen D., Xie F., Li Y. (2014). A nodule-specific lipid transfer protein AsE246 participates in transport of plant-synthesized lipids to symbiosome membrane and is essential for nodule organogenesis in Chinese milk vetch. Plant Physiol..

[32] Si Z., Guan N., Zhou Y., Mei L., Li Y., Li Y. (2020). A methionine sulfoxide reductase B is required for the establishment of Astragalus sinicus-Mesorhizobium symbiosis. Plant Cell Physiol..

[33] Zhou D., Li Y., Wang X., Xie F., Chen D. (2019). *Mesorhizobium huakuii HtpG Interaction with nsLTP AsE*246 Is Required for Symbiotic Nitrogen Fixation. Plant Physiol..

[34] Wang S., Hao B., Li J., Gu H., Peng J., Xie F., Zhao X., Frech C., Chen N., Ma B. (2014). Whole-genome sequencing of Mesorhizobium huakuii 7653R provides molecular insights into host specificity and symbiosis island dynamics. BMC Genom..

[35] Li Y., Zhou L., Li Y., Chen D., Tan X., Lei L., Zhou J. (2008). A nodule-specific plant cysteine proteinase, AsNODF32, is involved in nodule senescence and nitrogen fixation activity of the green manure legume *Astragalus sinicus*. New Phytol..

[36] Marx C.J., Lidstrom M.E. (2002). Broad-host-range cre-lox system for antibiotic marker recycling in gram-negative bacteria. BioTechniques.

[37] Kovach M.E., Elzer P.H., Hill D.S., Robertson G.T., Farris M.A., Roop R.M., Peterson K.M. (1995). Four new derivatives of the broad-host-range cloning vector pBBR1MCS, carrying different antibiotic-resistance cassettes. Gene.

[38] Van den Eede G., Deblaere R., Goethals K., Van Montagu M., Holsters M. (1992). Broad host range and promoter selection vectors for bacteria that interact with plants. Mol. Plant-Microbe Interact..

[39] Wilson K.J., Sessitsch A., Corbo J.C., Giller K.E., Akkermans A.D., Jefferson R.A. (1995). beta-Glucuronidase (GUS) transposons for ecological and genetic studies of rhizobia and other gram-negative bacteria. Microbiology.

[40] Stuurman N., Pacios Bras C., Schlaman H.R., Wijfjes A.H., Bloemberg G., Spaink H.P. (2000). Use of green fluorescent protein color variants expressed on stable broad-host-range vectors to visualize rhizobia interacting with plants. Mol. Plant-Microbe Interact..

[41] Hardy R., Burns R.C., Holsten R.D. (1973). Applications of the acetylene-ethylene assay for measurement of nitrogen fixation. Soil Biol. Biochem..

[42] Si Z., Yang Q., Liang R., Chen L., Chen D., Li Y. (2019). Digalactosyldiacylglycerol synthase gene MtDGD1 plays an essential role in nodule development and nitrogen fixation. Mol. Plant-Microbe Interact..

[43] Tamura K., Stecher G., Kumar S. (2021). MEGA11: Molecular Evolutionary Genetics Analysis Version 11. Mol. Biol. Evol..

[44] Pegues J.C., Chen L.S., Gordon A.W., Ding L., Coleman W.G. (1990). Cloning, expression, and characterization of the *Escherichia coli* K-12 rfaD gene. J. Bacteriol..

[45] Loutet S.A., Flannagan R.S., Kooi C., Sokol P.A., Valvano M.A. (2006). A complete lipopolysaccharide inner core oligosaccharide is required for resistance of *Burkholderia cenocepacia* to antimicrobial peptides and bacterial survival in vivo. J. Bacteriol..

[46] Walker L., Lagunas B., Gifford M.L. (2020). Determinants of host range specificity in legume-rhizobia symbiosis. Front. Microbiol..

[47] Kneidinger B., Marolda C., Graninger M., Zamyatina A., Mcarthur F., Kosma P., Valvano M.A., Messner P. (2002). Biosynthesis Pathway of ADP-L-glycero-beta-D-manno-Heptose in *Escherichia coli*. J. Bacteriol..

[48] Wang J., Ma W., Wang Z., Li Y., Wang X. (2014). Construction and characterization of an *Escherichia coli* mutant producing Kdo₂-lipid A. Mar. Drugs.

[49] Tang G., Wang Y., Luo L. (2014). Transcriptional regulator LsrB of Sinorhizobium meliloti positively regulates the expression of genes involved in lipopolysaccharide biosynthesis. Appl. Environ. Microbiol..

[50] Wakao S., Siarot L., Aono T., Oyaizu H. (2015). Effects of alteration in LPS structure in Azorhizobium caulinodans on nodule development. J. Gen. Appl. Microbiol..

[51] Kannenberg E.L., Carlson R.W. (2001). Lipid A and O-chain modifications cause Rhizobium lipopolysaccharides to become hydrophobic during bacteroid development. Mol. Microbiol..

[52] Noel K.D., Box J.M., Bonne V.J. (2004). 2-O-methylation of fucosyl residues of a rhizobial lipopolysaccharide is increased in response to host exudate and is eliminated in a symbiotically defective mutant. Appl. Environ. Microbiol..

[53] D’Haeze W., Leoff C., Freshour G., Noel K.D., Carlson R.W. (2007). Rhizobium etli CE3 bacteroid lipopolysaccharides are structurally similar but not identical to those produced by cultured CE3 bacteria. J. Biol. Chem..

[54] Ferguson B.J., Indrasumunar A., Hayashi S., Lin M.H., Lin Y.H., Reid D.E., Gresshoff P.M. (2010). Molecular analysis of legume nodule development and autoregulation. J. Integr. Plant Biol..

[55] Hastwell A.H., Corcilius L., Williams J.T., Gresshoff P.M., Payne R.J., Ferguson B.J. (2019). Triarabinosylation is required for nodulation-suppressive CLE peptides to systemically inhibit nodulation in Pisum sativum. Plant Cell Environ..

[56] Simon R., Priefer U., Puhler A. (1983). A broad host mobilization system for in vivo genetic engineering: Transposon mutagenesis in Gram-negative bacteria. Bio/Technolgy.

