# Supplementary material for: A Lipopolysaccharide Synthesis Gene rfaD from Mesorhizobium huakuii Is Involved in Nodule Development and Symbiotic Nitrogen Fixation
Source: Microorganisms. 2022 Dec 25;11(1):59. doi: 10.3390/microorganisms11010059 (PMC9866225; doi:10.3390/microorganisms11010059)
Supplement: Supplementary file 1 [file microorganisms-11-00059-s001.zip › microorganisms-2093877-supplementary.pdf]

microorganisms

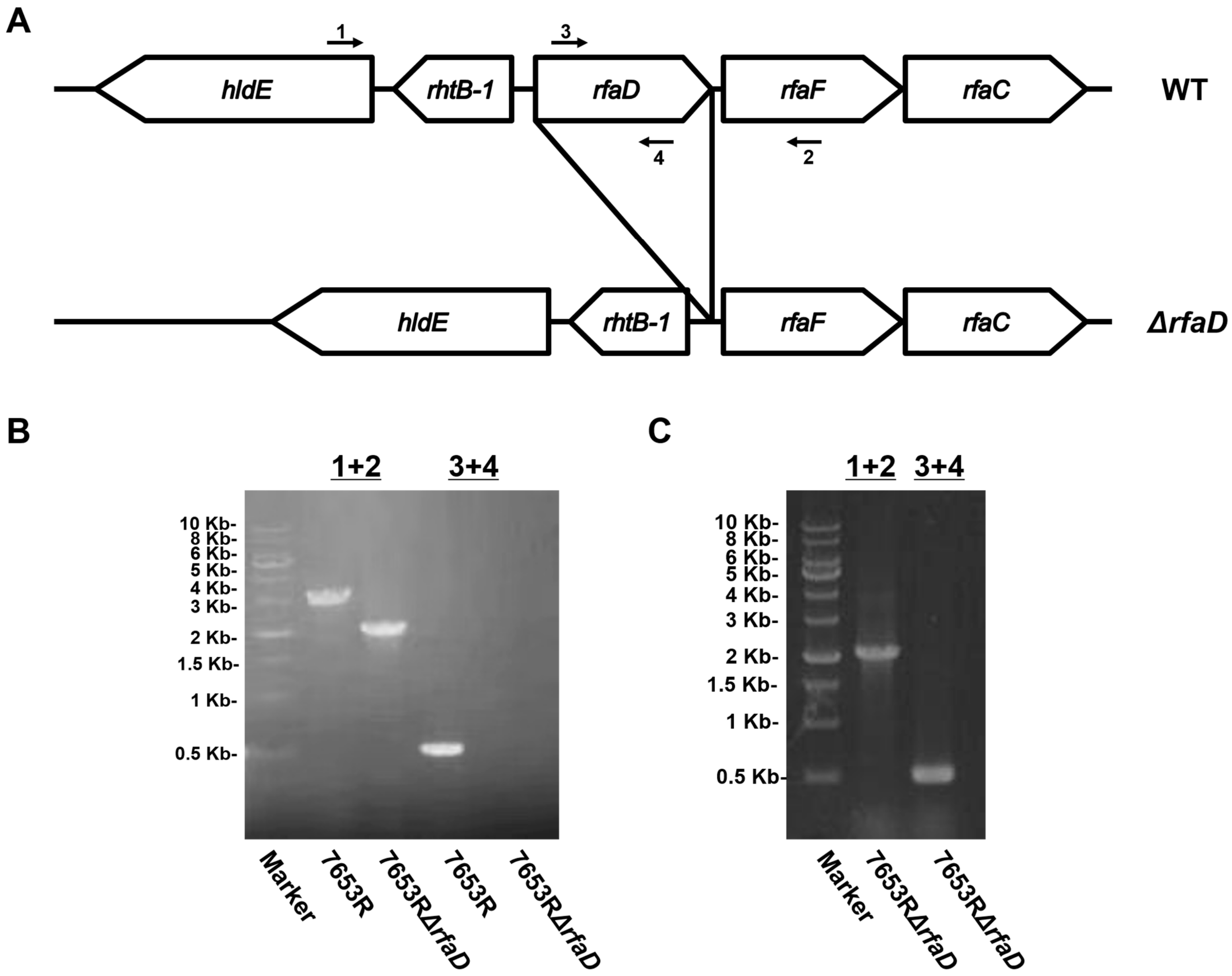

Figure S1 Verification of the constructed *M. huakuii* 7653R *rfaD* deletion mutant (7653R*ΔrfaD*). (**A**), Genomic organization of the wild-type 7653R (WT; *M. huakuii* 7653R) and the *ΔrfaD* mutant. (**B**), The *ΔrfaD* mutant was confirmed by PCR. (**C**). The *ΔrfaD* complementary strain was confirmed by PCR. The numbers 1 and 4 represent primers rfaD-Map-F and rfaD-Map-R, while numbers 2 and 3 represent primers rfaD-ORF-YF and rfaD-ORF-YR, respectively.

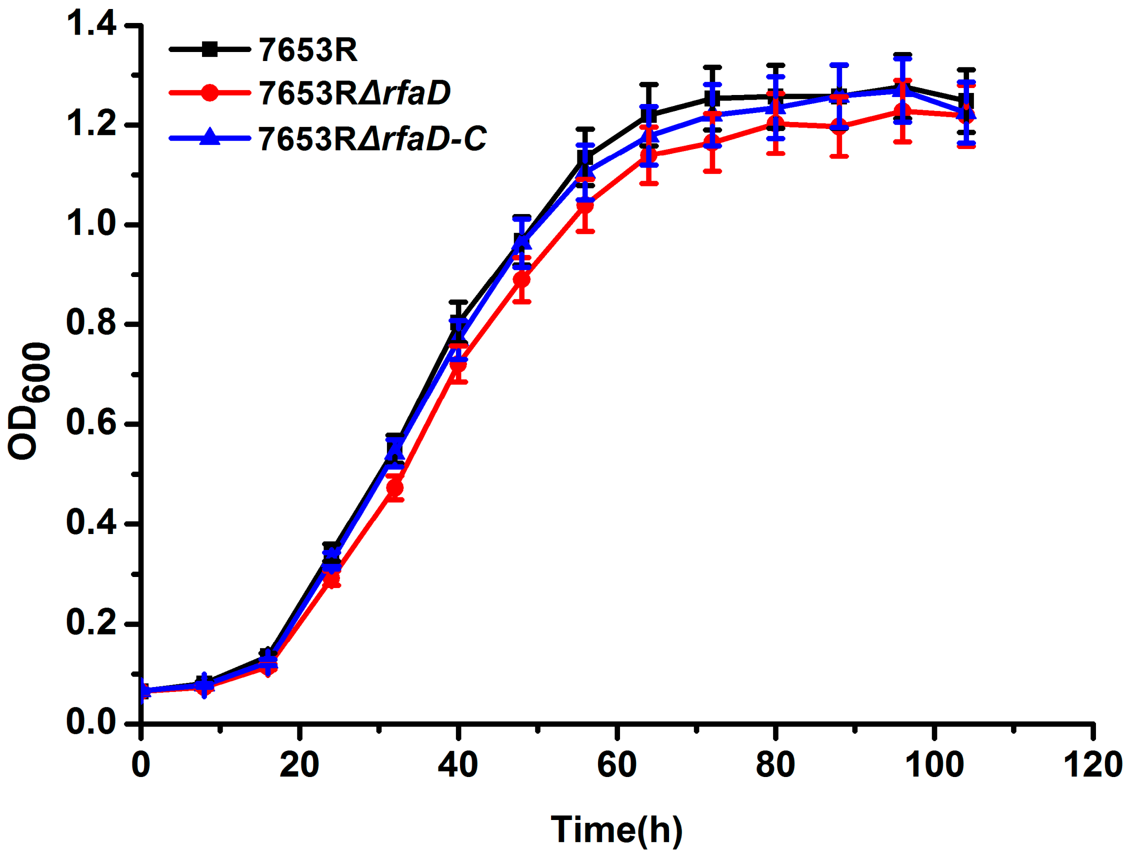

Figure S2 Effects of 7653R *rfaD* on the rhizobia growth under free-living conditions. Growth curves of the wild-type 7653R, 7653RΔ*rfaD* and 7653RΔ*rfaD*-C. All of the strains were grown in liquid TY medium. The error bars represent the variant ranges of the data of three biological replicates.

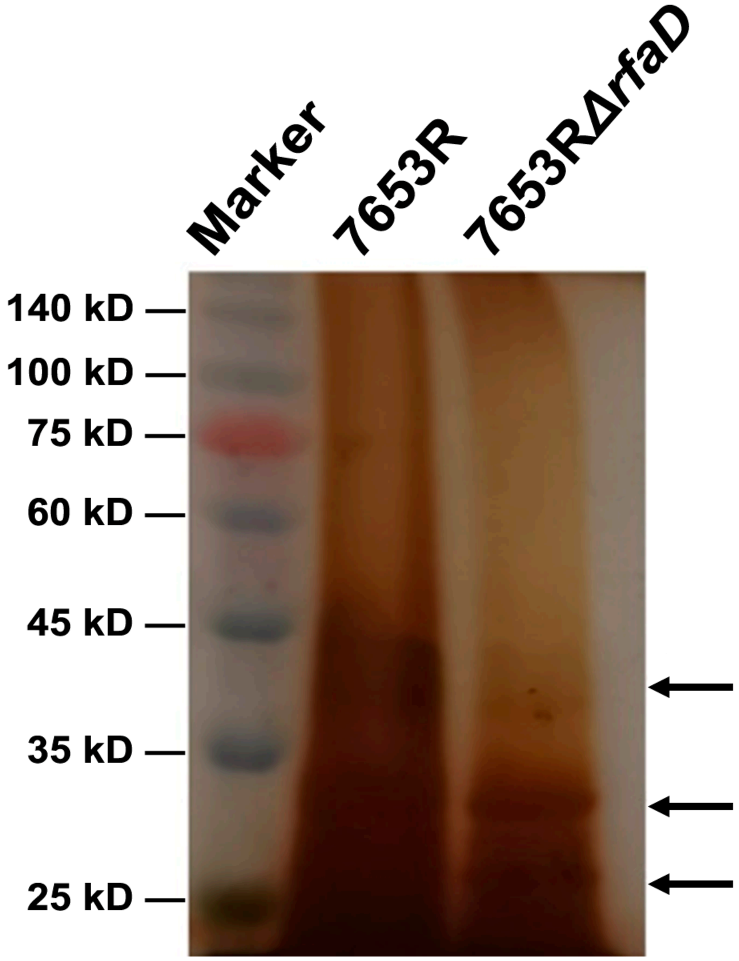

Figure S3 The LPS produced by wild-type 7653R and 7653RΔ*rfaD* mutant. The LPS were extracted from wild-type 7653R and 7653RΔ*rfaD* strains that were cultured in the TY liquid medium (OD_{600} to 1.0) with the LPS extraction kit according to the manufacturer's instructions (iNtRON). The samples were separated by SDS-PAGE and then stained by silver solution.

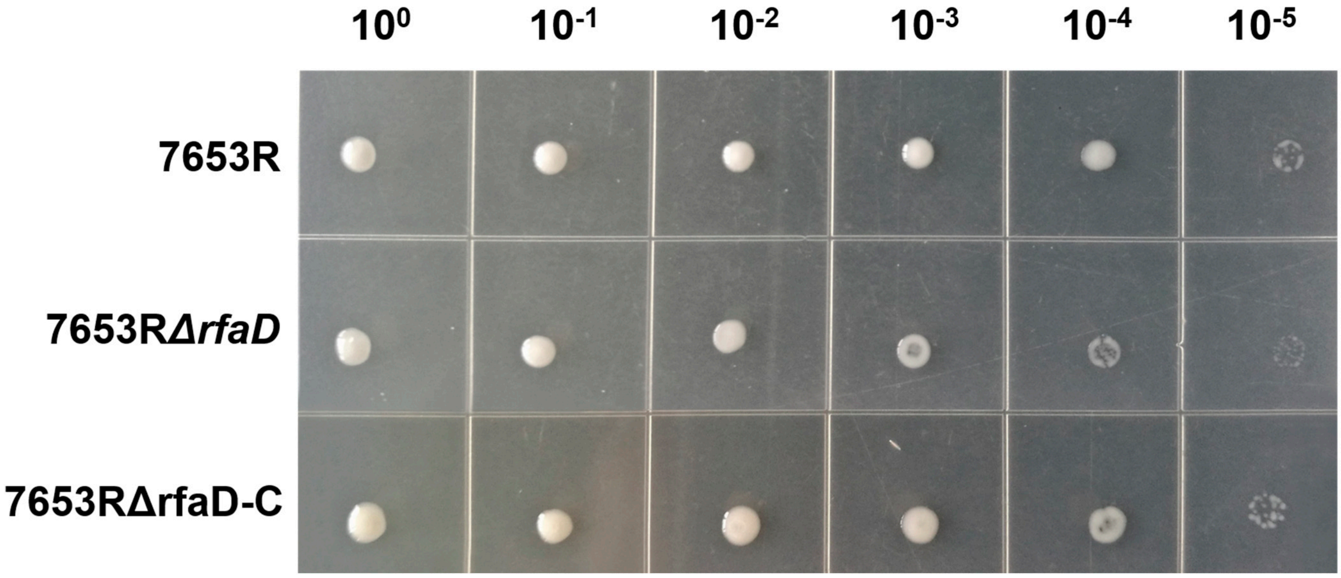

Figure S4 Sensitivity of the 7653R, 7653RΔrfaD and 7653RΔrfaD-C strains to the polymyxin B. Wild-type 7653R, 7653RΔ*rfaD* and 7653RΔ*rfaD*-C were firstly grown in the TY liquid medium (OD_{600} to 1.0). Subsequently, the bacterial cells were collected and washed by sterile water, then resuspended (OD_{600} to 1.0). A series of 10×dilutions were spotted on the TY plates added with 50 μg/ml polymyxin B.

Table S1. The strains and plasmids used in this study

Strain or plasmid	Relevant characteristics	Reference or source
E. coli		
DH5α	*supE44 ΔlacU169(φ80lacZΔM15) hsdR17 recA1 endA1 gyrA96 thi-1 relA1*	TaKaRa
S17-1	*recA*, harbours the *tra*genes of plasmid RP4 in the chromosome; *proA*, *thi-1*	[1]
M. huakuii		
7653R	Wild type, Nod^+, Fix^+, Str^r	This study
7653R-GFP	Wild type 7653R harboring the pMP2463 vector, Str^r, Gm^r	This study
ΔrfaD	Deletion mutantof the *rfaD* gene, Str^r	This study
ΔrfaD-C	Complementation of *ΔrfaD* by harboring the pBBR1MCS-5-*rfaD*, Str^r, Gm^r	This study
7653R*ΔrfaD* -GFP	*ΔrfaD* harboring the pMP2463 vector, Str^r, Gm^r	This study
**Plasmids**		
pRG960	pRG930 containing the promoterless*gusA*with the start codon, Sp^r	[2]
pCM351	Allelic exchange vector, Gm^r	[3]
pCM157	The *cre* expression vector,Tc^r	[3]
pBBR1MCS-5	Broad host-range expression vector, Gm^r	[4]
pMP2463	pBBR1MCS5 derivate, Gm^r	[5]

Table S2. Primers used in this study

Name	Sequence 5′→ 3′	Usage
rfaD-Pro-F	AAAACTGCAGCCGGGGATGTAGGCGAAGAT	Promoter-GUS analysis
rfaD-Pro-R	CGCGGATCCGGCCTCTGGTTGCTTGCG	Promoter-GUS analysis
rfaD-up-F	GGGGTACCCTTCTGCAGGGCAACGT	Homologous fragment
rfaD-up-R	GCGCATATGCATGAAGGCCTCTGGTTG	Homologous fragment
rfaD-down-F	TGGGCCCCCCAACGATCCTCGTGA	Homologous fragment
rfaD-down-F	GACCGGTGTAGACGGCGACTATCGG	Homologous fragment
rfaD-Map-F	CCATGCAGCACGGGAAT	Validation of *rfaD* mutation
rfaD-Map-R	TCGACGCACCAGTCGAAG	Validation of *rfaD* mutation
loxp-F	CAGGGTTATTGTCTCATGAGCGG	Validation of *rfaD* mutation
loxp-R	CGACGCTCGAACGGGACTAC	Validation of *rfaD* mutation
Gm-F	ATGTTACGCAGCAGCAACG	Cloningthe ORF of Gm
Gm-R	TTAGGTGGCGGTACTTGGG	Cloningthe ORF of Gm
rfaD-ORF-YF	CGGCAAGTTCATGATGGAGGTG	Validation of *rfaD* mutation
rfaD-ORF-YR	CGCGCAAACGGGACATATCA	Validation of *rfaD* mutation
pBBR5-*rfaD*-F	CACGCGTCGACCCGGGGATGTAGGCGAAGAT	Complemental fragment
pBBR5-*rfaD*-R	CCGGAATTCGCACTGCGCCATCACCAT	Complemental fragment
M13-F	GAGCGGATAACAATTTCACACAGG	Validation of *rfaD* mutation
M13-R	CGCCAGGGTTTTCCCAGTCACGAC	Validation of *rfaD* mutation

Restriction enzyme sites are underlined.

Supplemental Reference

1. Simon, R.; Priefer, U.; Puhler, A. A broad host mobilization system for in vivo genetic engineering: Transposon mutagenesis in Gram-negative bacteria. *Bio/Technolgy* **1983**, *1*, 37-45.
2. Van den Eede, G.; Deblaere, R.; Goethals, K.; Van Montagu, M.; Holsters, M. Broad host range and promoter selection vectors for bacteria that interact with plants. *Molecular Plant-Microbe Interactions : MPMI* **1992**, *5*, 228-234, doi:10.1094/mpmi-5-228.
3. Marx, C.J.; Lidstrom, M.E. Broad-host-range cre-lox system for antibiotic marker recycling in gram-negative bacteria. *BioTechniques* **2002**, *33*, 1062-1067, doi:10.2144/02335rr01.
4. Kovach, M.E.; Elzer, P.H.; Hill, D.S.; Robertson, G.T.; Farris, M.A.; Roop, R.M., 2nd; Peterson, K.M. Four new derivatives of the broad-host-range cloning vector pBBR1MCS, carrying different antibiotic-resistance cassettes. *Gene* **1995**, *166*, 175-176, doi:10.1016/0378-1119(95)00584-1.
5. Stuurman, N.; Pacios Bras, C.; Schlaman, H.R.; Wijfjes, A.H.; Bloemberg, G.; Spaink, H.P. Use of green fluorescent protein color variants expressed on stable broad-host-range vectors to visualize rhizobia interacting with plants. *Molecular Plant-Microbe Interactions : MPMI* **2000**, *13*, 1163-1169, doi:10.1094/mpmi.2000.13.11.1163.
